# Efficient Two-Dimensional Direction Finding Algorithm for Rectilinear Sources Under Unknown Mutual Coupling

**DOI:** 10.3390/s20071914

**Published:** 2020-03-30

**Authors:** Jian Xie, Qiuping Wang, Yuexian Wang, Xin Yang

**Affiliations:** Electronics and Information School, Northwestern Polytechnical University, Xi’an 710072, China; xiejian@nwpu.edu.cn (J.X.); wangqiuping.npu@mail.nwpu.edu.cn (Q.W.); xinyang@nwpu.edu.cn (X.Y.)

**Keywords:** DOA estimation, mutual coupling, rectilinear sources, RARE.

## Abstract

Digital communication signals in wireless systems may possess noncircularity, which can be used to enhance the degrees of freedom for direction-of-arrival (DOA) estimation in sensor array signal processing. On the other hand, the electromagnetic characteristics between sensors in uniform rectangular arrays (URAs), such as mutual coupling, may significantly deteriorate the estimation performance. To deal with this problem, a robust real-valued estimator for rectilinear sources was developed to alleviate unknown mutual coupling in URAs. An augmented covariance matrix was built up by extracting the real and imaginary parts of observations containing the circularity and noncircularity of signals. Then, the actual steering vector considering mutual coupling was reparameterized to make the rank reduction (RARE) property available. To reduce the computational complexity of two-dimensional (2D) spectral search, we individually estimated *y*-axis and *x*-axis direction-cosines in two stages following the principle of RARE. Finally, azimuth and elevation angle estimates were determined from the corresponding direction-cosines respectively. Compared with existing solutions, the proposed method is more computationally efficient, involving real-valued operations and decoupled 2D spectral searches into twice those of one-dimensional searches. Simulation results verified that the proposed method provides satisfactory estimation performance that is robust to unknown mutual coupling and close to the counterparts based on 2D spectral searches, but at the cost of much fewer calculations.

## 1. Introduction

Uniform rectangular array (URA), due to its versatility in providing both azimuthal and elevational coverage simultaneously, has attracted much attention in many applications, such as radar, sonar, and wireless communication [1]. Two-dimensional (2D) direction finding with URA is thus of practical use in many situations. Based on the subspace principle, the classical 1D super resolution algorithms such as multiple signal classification (MUSIC) [2] and compressive sensing [3] can be readily generalized to URA. Moreover, to further reduce the computational complexity or increase the accuracy of the direction finding results, some effective schemes have been proposed [4,5,6,7,8]. In [4], the authors proposed a 2D unitary ESPRIT algorithm for URA, which can be realized in both element space and beamspace. Also, its main procedure is formulated in terms of real-valued operation and is therefore computationally efficient. In [5], a dual-size spatial invariance array structure was proposed. Based on the ESPRIT theory, it utilizes unambiguous but low-accuracy estimates to disambiguate high-accuracy but periodically ambiguous direction cosine estimates. In [6], a 2D matrix pencil method is developed to convert the complex data matrix to a real one, which can reduce the complexity of the computation significantly. In [7], to relieve the computational burden involved in 2D spectral searches, the authors decoupled the 2D MUSIC problem into two successive real-valued one-dimensional (1D) DOA estimation problems. In [8], a 2D unitary ESPRIT-like DOA estimation algorithm was developed for coherent signals based on a URA. It can provide better decorrelation performance and closed-form solutions due to the properties of the 2D ESPRIT-like method.

Recently, in modern wireless communication systems, rectilinear or strictly noncircular signals such as amplitude modulated (AM), binary phase shift keying (BPSK), and pulse-amplitude modulated (PAM) signals are commonly applied. Notice that both conjugated and unconjugated covariance matrices of rectilinear signals include the second-order statistical information [9]. Hence, the rectilinearity can be utilized to promote the estimation accuracy and increase the maximum resolvable sources. Accordingly, various algorithms have been developed to fully exploit the rectilinearity for DOA estimation performance enhancement. In [10], Abeida and Delmas proposed a tailored MUSIC algorithm for noncircular sources called NCMUSIC, which combines the statistics of conjugated and unconjugated covariance matrices and thus outperforms the standard MUSIC algorithm. In [11], the sparse representation technique was introduced for representing conjugated and unconjugated covariance jointly with a sparsity constraint, which surpasses the performance of NCMUSIC. However, it is computationally demanding since it requires complicate convex optimization procedures. In [12], the difference and sum coarray was utilized for the DOA estimation of noncircular quasi-stationary sources, which can extend the degrees of freedom (DOF) of the array effectively. In [13,14], the scenario of coexisting circular and noncircular sources is considered, and various algorithms have been further developed to distinguish the mixed sources and estimate the DOA parameters. Based on an URA, the authors proposed a root-MUSIC method to obtain the 2D DOA parameters and classification of the mixed sources [15]. In [16], the authors proposed a localization algorithm for rectilinear sources based on the rank reduction (RARE) principle, which can cope with the mixed near-field and far-field sources.

Generally, the above mentioned DOA estimation algorithms are based on the assumption that the array manifold is ideal without any perturbation. However, in practical direction finding systems, systematic errors such as mutual coupling, gain and phase errors, and sensor position errors are inevitable [17]. These model mismatches would substantially degrade the DOA estimation performance of these algorithms. Mutual coupling effect, as an electromagnetic interaction between the array elements, causes the current distribution of each sensor to depend on the excitations from both their own and other adjacent element. It deviates the signal subspace of the observed array covariance matrix from the ideal array steering vector, thus causing a decrease in direction finding performance. Therefore, the mutual coupling effect cannot be ignored in the development of these algorithms.

Fortunately, many effective schemes have been proposed by various researchers to compensate for the mutual coupling effect or to obtain the DOA estimates blindly. One class of compensation methods are based on calibration sources. Using a set of predefined sources in known locations, the mutual coupling coefficients can be calculated by the least square method [18]. However, the calibration sources are often inaccessible in real-time direction finding applications. Moreover, the mutual coupling characteristics of the array may vary as the environmental conditions change, and therefore the calibration source-based methods are only effective under certain circumstances. Consequently, another class of auto-calibration algorithms is advanced to compensate for the mutual coupling effect without any calibration sources [19,20,21,22,23,24,25]. In [19], an eigenstructure-based algorithm was developed for the DOA estimation and mutual coupling auto-calibration at the same time. However, it requires multidimensional alternating minimization which is computationally prohibitive. To avoid exhaustive multidimensional searches over the unknown DOA and mutual coupling parameters, the authors proposed a modified MUSIC algorithm [20]. By taking the sensors at either side of the array as auxiliary ones, the authors found that the MUSIC algorithm is resilient against unknown mutual coupling effects. However, the array aperture loss problem of these methods is severe particularly under the strong mutual coupling scenario, because only the observations from middle subarray array elements can be utilized for DOA estimation. Aiming at resolving this problem, Liao et al. utilized the banded symmetric Toeplitz property of the mutual coupling matrix and transformed this matrix into gain and phase perturbations of the array [21], where the perturbations of the middle subarray elements are identical. Consequently, the observation data from all the array elements can be fully utilized for DOA estimation via simple 1D RARE method. Furthermore, a fourth order cumulant (FOC) method was also proposed [22], which performs better especially for strong mutual coupling effects. In [23,24,25], the authors utilized sparse reconstruction methods to improve the DOA estimation performance under unknown mutual coupling. However, these methods are computationally demanding due to the *l*_1_-norm minimization problem. In [26], the authors presented a method for the localization of mixed sources using the noncircular information of the impinging signals under unknown mutual coupling. In [27], an ESPRIT-based algorithm is proposed for the ODA estimation of strictly noncircular sources with unknown mutual coupling, which has large virtual array aperture and low computational load. According to the latest work by B. Friedlander [28], the mutual coupling matrix is a direction dependent matrix, and not a constant matrix as is commonly assumed, which may impact some of the work presented in the DOA estimation literature. However, few of the works on DOA estimation have taken into account the direction-dependent mutual coupling effect. In [29], a decoupled 2D DOA estimation algorithm is proposed for uniform circular arrays. Yet, it assumes that the mutual coupling effect is only elevation-dependent. Also, it is an offline technique, for which is costly to build a calibration setup. In [30], a unified transformation method is proposed for the DOA estimation under direction-dependent mutual coupling. This method can be used in many array structures, but it requires convex optimization and iteration processes, which is computationally inefficient. In [31], the authors proposed a RARE-based low complexity DOA estimation method to solve the direction-dependent mutual coupling problem. Moreover, coprime sparse arrays are proposed to reduce the mutual coupling effect between the elements [32,33,34,35].

For the 2D array calibration problem, several effective methods have been developed [36,37,38,39,40]. In [36], the authors proposed a blind calibration scheme for uniform circular array based on azimuthal symmetric structures in the mutual coupling matrix. However, it only provides the azimuth angle estimates of the radiating sources, while the elevation angles are assumed to be zeros. In [37], a robust method was proposed to calibrate the mutual coupling and channel gain/phase inconsistency of uniform circular array. Yet, it requires calibration sources and 2D spectral searches. In [38], the auxiliary sensor-based algorithm is generalized for URA and uniform hexagon arrays, where the elements at the boundary of the array are set as the auxiliary ones. In order to mitigate the array aperture loss problem in the auxiliary sensor-based method, Wu et al. proposes a RARE-based auto-calibration method for URA [40], which can fully utilize the array observations. However, all these planar array calibration methods require exhaustive 2D spectral searches for the DOA estimation, which is computationally demanding.

To the extent of our knowledge, few works have been devoted to the 2D direction finding algorithms for rectilinear sources in the presence of unknown mutual coupling effects. Despite the various advantages that were manifested in the approaches cited above, there are some fundamentally inherent problems to be overcome in view of the previous analyses: (1) all of the above-mentioned 2D DOA estimation works only use the covariance matrix for direction finding and mutual coupling calibration, the rectilinearity of the sources are not fully utilized. (2) These works require 2D spectral searches for the azimuth and elevation angle estimations, which is computationally demanding. (3) Generally, subspace-based direction finding algorithms require complex-valued operations for the calculation of covariance matrix, eigenvalue decomposition (EVD), and spectral searching. Many works have been devoted to converting the complex-valued covariance into a real-valued one. The computational complexity of EVD and spectral searches for this kind of transformation can be decreased by a factor of four. Yet, the transformation itself and the computation of the covariance still require additional complex-valued operations.

Therefore, aiming at addressing these problems, we developed a computationally efficient 2D direction finding algorithm for rectilinear signals under unknown mutual coupling. Based on the rectilinearity of the signal, the received data is transformed into the real-valued domain. Then the structure of the virtual steering vector is utilized to decouple the DOA parameter from other nuisance parameters. According to the RARE principle, the *y*-axis direction-cosine estimates are first obtained via a 1D spectral search. Consequently, the *x*-axis direction-cosines are then generated through another RARE estimator, which also only requires a 1D spectral search with the *y*-axis direction-cosine estimates.

The main contributions of this paper are as follows:

(1) The proposed DOA estimation method avoids the two-dimensional spectral search and effectively reduces the computational complexity;
(2) Only real-valued operations are needed, and its computation load is reduced to 1/4 of the complex value operations;
(3) The estimation accuracy is improved by the efficient use of the source rectilinearity.

The rest of this paper is organized as follows. In Section 2, we present the signal model for the rectilinear sources of a URA under unknown mutual coupling. In Section 3, derivation of the proposed real-valued two stage 2D DOA estimation algorithm is detailed. Then we discuss the computational complexity and the maximum number of resolvable sources in Section 4. In Section 5, several numerical simulations are carried out to validate the performance of the proposed algorithm. In Section 6, the paper ends with conclusions. Finally, in the Appendix A, we have derived the Cramér-Rao Bound (CRB) for the problem. 

Throughout the paper, we make use of the following notations as listed in Table 1.

## 2. Signal Model

**Figure 1:**
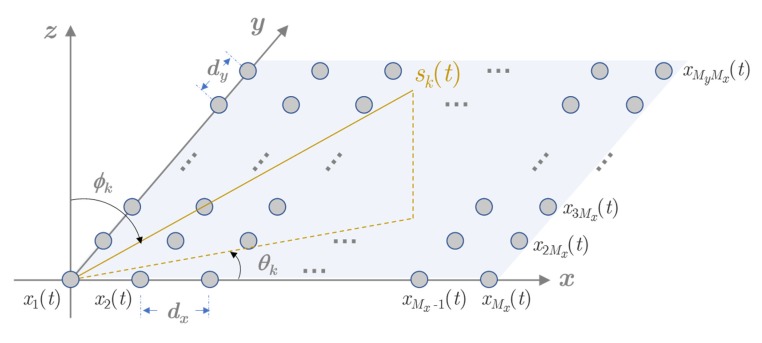
The structure of a uniform rectangular array (URA) with *M* elements.

As shown in Figure 1, consider a URA composed of *M* = *M_x_* × *M_y_* omnidirectional elements, with the inter element spacing along the *x*-axis and the *y*-axis being *d_x_* and *d_y_*, respectively. Suppose that there are *K* far-field narrowband sources radiating onto the URA from directions $\left(\theta_{1},\phi_{1}\right)$, …,$\left(\theta_{K},\phi_{K}\right)$, where $\theta_{k}$ and $\phi_{k}$ are the azimuth and elevation angles of the *k*-th source.

Under the ideal scenario, the received data of each element only depends on the incident wavefield. Accordingly, the ideal array received data vector without mutual coupling can be modeled as

(1) $$x(t)=\sum_{k=1}^{K}a(\theta_{k},\phi_{k})s_{k}(t)+n(t)=As(t)+n(t)$$

where $x(t)={\left[x_{1}(t),\cdots ,x_{M_{x}}(t),x_{M_{x}+1}(t),\cdots ,x_{2M_{x}}(t),\cdots ,x_{M_{y}M_{x}}(t)\right]}^{T}$, $s(t)={\left[s_{1}(t),s_{2}(t),\cdots ,s_{K}(t)\right]}^{T}$ is the source signal vector, $n(t)={\left[n_{1}(t),\cdots ,n_{M_{x}}(t),n_{M_{x}+1}(t),\cdots ,n_{2M_{x}}(t),\cdots ,n_{M_{y}M_{x}}(t)\right]}^{T}$ denotes the array noise vector, and $A=[a(\theta_{1},\phi_{1}),\cdots ,a(\theta_{K},\phi_{K})]$ represents the ideal steering matrix, with its steering vectors being

(2) $$a(\theta_{k},\phi_{k})=a_{y}(\psi_{y,k})⊗a_{x}(\psi_{x,k}),\;k=1,\cdots ,K$$

where

(3) $$a_{x}(\psi_{x,k})={\left[1,e^{j\psi_{x,k}},\cdots ,e^{j(M_{x}-1)\psi_{x,k}}\right]}^{T}$$

(4) $$a_{y}(\psi_{y,k})={\left[1,e^{j\psi_{y,k}},\cdots ,e^{j(M_{y}-1)\psi_{y,k}}\right]}^{T}$$

Herein, $\psi_{x,k}$ and $\psi_{y,k}$ are the electrical angles of the kth source with respect to the *x*-axis and the *y*-axis, with $\psi_{x,k}=2\pi d_{x}\cos\theta_{k}\sin\phi_{k}/\lambda$ and $\psi_{y,k}=2\pi d_{y}\sin\theta_{k}\sin\phi_{k}/\lambda$ where *λ* is the carrier wavelength.

However, in practical direction finding systems, a mismatch may be introduced by the electromagnetic interaction between the array elements. Therefore, taking into account the mutual coupling effect between the array elements, we can modify the received data model as

(5) x(t)=CAs(t)+n(t)

where **C** is the $M_{x}M_{y}\times M_{x}M_{y}$ mutual coupling matrix, with its elements being mutual coupling coefficients (MCCs). As illustrated in [19], the mutual coupling effect between the elements is inversely proportional to their distance. Thus, the MCCs between the neighboring sensors are approximately equal. When two elements are far apart, the mutual coupling effect is negligible, and the coefficients approach zero. As in [30], the general mutual coupling model for URA is developed by the following block matrix

(6) $$C=\left[\begin{array}{ccccccc} C_{1}^{} & C_{2}^{} & \cdots & C_{P}^{} & & & \\ C_{2}^{} & C_{1}^{} & C_{2}^{} & & \ddots & & \\ \vdots & \ddots & & \ddots & & \ddots & \\ C_{P}^{} & & C_{2}^{} & C_{1}^{} & C_{2}^{} & & C_{P}^{}\\ & \ddots & & \ddots & \ddots & \ddots & \vdots \\ & & \ddots & & C_{2}^{} & C_{1}^{} & C_{2}^{}\\ & & & C_{P}^{} & \cdots & C_{2}^{} & C_{1}^{} \end{array}\right]$$

and the $M_{x}\times M_{x}$ submatrix **C***_p_* (*p* = 1,…,*P*) is expressed as [27]

(7) $$C_{p}=\left[\begin{array}{cccccc} c_{p-1,0} & c_{p-1,1} & \cdots & c_{p-1,P-1} & & \\ c_{p-1,1} & c_{p-1,0} & c_{p-1,1} & & \ddots & \\ \vdots & c_{p-1,1} & c_{p-1,0} & \ddots & & c_{p-1,P-1}\\ c_{p-1,P-1} & & \ddots & \ddots & c_{p-1,1} & \vdots \\ & \ddots & & c_{p-1,1} & c_{p-1,0} & c_{p-1,1}\\ & & c_{p-1,P-1} & \cdots & c_{p-1,P-1} & c_{p-1,0} \end{array}\right]$$

The complex mutual coupling coefficient $c_{m,n}=\rho_{m,n}e^{j\xi_{m,n}}$ symbolizes the electromagnetic interaction between a reference sensor defined as the origin of a Cartesian coordinate system and the sensor located at (±*md_x_*, ±*nd_y_*) under the corresponding coordinate [30]. Note that $c_{0,0}$ is especially defined as 1. Therefore, the mutual coupling matrix **C** can be further expressed as

(8) $$C=\sum_{p=1}^{P}J_{p}⊗C_{p}$$

and $J_{p}$ is a $M_{y}\times M_{y}$ selection matrix, whose elements are

(9) $${\left[J_{p}\right]}_{m,n}=\{\begin{array}{l} 1,\;\left|m-n\right|=p-1\\ 0,\;\mathrm{otherwise} \end{array}$$

Moreover, for rectilinear sources, the signal vector is of the following form [10]

(10) $$s(t)=\Phi s_{0}^{}(t)$$

where $s_{0}^{}(t)={\left[s_{0,1}^{}(t),\cdots ,s_{0,K}^{}(t)\right]}^{T}$, with $s_{0,k}(t)$ being the real-valued zero-phase version of the *k*-th source signal, $\Phi =\mathrm{diag}\text{\{}e_{}^{j\varphi_{1}},\cdots ,e_{}^{j\varphi_{K}}\text{\}}$ contains the noncircular phase shifts of the sources [9].

Owing to the above analysis, the received data vector of the planar array can be formulated as

(11) $$x(t)=CA\Phi s_{0}(t)+n(t)$$

With the received data vector **x**(*t*), the main problem to be addressed in this paper is to obtain the 2D DOA estimates of rectilinear signals under unknown mutual coupling effects. Unlike the traditional methods which require multidimensional spectral searches and omit the rectilinearity of the signals, a novel algorithm is investigated in the next section for reducing the computational complexity and promoting the estimation performance.

## 3. Proposed Real-Valued 2D DOA Estimation Algorithm

In order to generate accurate 2D DOA estimates with low computational complexity, we creatively utilize the rectilinearity of signals and factorize the steering vector with respect to the DOA and other nuisance parameters. Our technique proceeds through the following stages: (1) based on the rectilinearity of the signal, the received data is transformed into the real-valued domain; (2) the structure of the virtual steering vector is utilized to decouple the DOA parameter from other nuisance parameters; (3) according to the RARE principle, the *y*-axis direction-cosine estimates can be obtained via a 1D spectral search; (4) with the *y*-axis direction-cosine estimates, the *x*-axis direction-cosine can be found through another RARE estimator, which also only requires a 1D spectral search. The following subsections give the details of the proposed algorithm.

### 3.1. Extended Real-Valued Signal Model

It is well known that by transforming complex-valued data into the real-valued domain, the computational cost can be effectively reduced by a factor of four. Therefore, it inspired us to look for a more efficient algorithm to reduce the complex-valued computational loads.

Based on the sources’ rectilinearity, the transformation can be realized by extracting the real and imaginary parts of **x**(*t*), respectively.

(12) xR(t)=Re[x(t)], xI(t)=Im[x(t)]

From Equation (11), $x_{R}(t)$ and $x_{I}(t)$ can be further expressed as

(13) $$x_{R}(t)=A_{R}s_{0}(t)+n_{R}(t)$$

(14) $$x_{I}(t)=A_{I}s_{0}(t)+n_{I}(t)$$

where $A_{R}=[v_{R}(\theta_{1},\phi_{1},\varphi_{1},C),\cdots ,v_{R}(\theta_{K},\phi_{K},\varphi_{K},C)]$ and $A_{I}=[v_{I}(\theta_{1},\phi_{1},\varphi_{1},C),\cdots ,v_{I}(\theta_{K},\phi_{K},\varphi_{K},C)]$ are the real-valued manifold matrices of $x_{R}(t)$ and $x_{I}(t)$. $n_{R}(t)$ and $n_{I}(t)$ symbolize the real and imaginary components of sensor noise vector n(t), respectively. Accordingly, the real-valued virtual steering vectors in $A_{R}$ is of the following form

(15) $$v_{R}(\theta_{k},\phi_{k},\varphi_{k},C)=\left[Ca(\theta_{k},\phi_{k})\cdot e^{j\varphi_{k}}+C^{*}a^{*}(\theta_{k},\phi_{k})\cdot e^{-j\varphi_{k}}\right]/2$$

Based on Equations (2) and (8), we can obtain the following equation from the Kronecker product property (A⊗B)(C⊗D)=(AC)⊗(BD) [41]

(16) $$Ca(\theta_{k},\phi_{k})=\left(\sum_{p=1}^{P}J_{p}⊗C_{p}\right)\left(a_{y}(\psi_{y,k})⊗a_{x}(\psi_{x,k})\right)=\sum_{p=1}^{P}\left(J_{p}a_{y}(\psi_{y,k})\right)⊗\left(C_{p}a_{x}(\psi_{x,k})\right)$$

Therefore, the real-valued virtual steering vector $v_{R}(\theta_{k},\phi_{k},\varphi_{k},C)$ can be further written as

(17) $$\begin{array}{c} v_{R}(\theta_{k},\phi_{k},\varphi_{k},C)=\sum_{p=1}^{P}\left(J_{p}a_{y,c}(\psi_{y,k})\right)⊗\left(\Gamma_{c,p}(\psi_{x,k})a_{x,c}(\psi_{x,k})\right)-\sum_{p=1}^{P}\left(J_{p}a_{y,s}(\psi_{y,k})\right)⊗\left(\Gamma_{c,p}(\psi_{x,k})a_{x,s}(\psi_{x,k})\right)\\ -\sum_{p=1}^{P}\left(J_{p}a_{y,c}(\psi_{y,k})\right)⊗\left(\Gamma_{s,p}(\psi_{x,k})a_{x,s}(\psi_{x,k})\right)-\sum_{p=1}^{P}\left(J_{p}a_{y,s}(\psi_{y,k})\right)⊗\left(\Gamma_{s,p}(\psi_{x,k})a_{x,c}(\psi_{x,k})\right) \end{array}$$

where

(18) $$a_{x,c}(\psi_{x,k})={\left[1,\cos(\psi_{x,k}),\cdots ,\cos\left((M_{x}-1)\psi_{x,k}\right)\right]}^{T}$$

(19) $$a_{x,s}(\psi_{x,k})={\left[0,\sin(\psi_{x,k}),\cdots ,\sin\left((M_{x}-1)\psi_{x,k}\right)\right]}^{T}$$

(20) $$a_{y,c}(\psi_{y,k})={\left[1,\cos(\psi_{y,k}),\cdots ,\cos\left((M_{y}-1)\psi_{y,k}\right)\right]}^{T}$$

(21) $$a_{y,s}(\psi_{y,k})={\left[0,\sin(\psi_{y,k}),\cdots ,\sin\left((M_{y}-1)\psi_{y,k}\right)\right]}^{T}$$

(22) $$\Gamma_{c,p}(\psi_{x,k})=h_{c}^{\left(p\right)}\cdot \mathrm{diag}\left[\alpha_{c,1}^{\left(p\right)},\cdots ,\alpha_{c,P-1}^{\left(p\right)},1,\cdots ,1,\beta_{c,1}^{\left(p\right)},\cdots ,\beta_{c,P-1}^{\left(p\right)}\right]$$

(23) $$\Gamma_{s,p}(\psi_{x,k})=h_{s}^{\left(p\right)}\cdot \mathrm{diag}\left[\alpha_{s,1}^{\left(p\right)},\cdots ,\alpha_{s,P-1}^{\left(p\right)},1,\cdots ,1,\beta_{s,1}^{\left(p\right)},\cdots ,\beta_{s,P-1}^{\left(p\right)}\right]$$

(24) $$h_{c}^{\left(p\right)}=\sum_{i=1-P}^{P-1}\rho_{p-1,\left|i\right|}\cos\left(\xi_{p-1,\left|i\right|}+\varphi_{k}+i\cdot 2\pi \lambda^{-1}\psi_{x,k}\right)$$

(25) $$h_{s}^{\left(p\right)}=\sum_{i=1-P}^{P-1}\rho_{p-1,\left|i\right|}\sin\left(\xi_{p-1,\left|i\right|}+\varphi_{k}+i\cdot 2\pi \lambda^{-1}\psi_{x,k}\right)$$

Note that $\Gamma_{c,p}(\psi_{x,k})$ and $\Gamma_{s,p}(\psi_{x,k})$ are $M_{x}\times M_{x}$ diagonal matrices comprising $M_{x}-2P+2$ ones between $\alpha_{c,P-1}^{\left(p\right)}$ and $\beta_{c,1}^{\left(p\right)}$ (also between $\alpha_{s,P-1}^{\left(p\right)}$ and $\beta_{s,1}^{\left(p\right)}$). $h_{c}^{\left(p\right)}$ and $h_{s}^{\left(p\right)}$ are two scalar parameters determined by the *x*-axis direction-cosine, rectilinearity phases, and MCCs. For p=1,2,⋯,P−1, we have

(26) $$\alpha_{c,q}^{\left(p\right)}=\frac{h_{c}^{\left(p\right)}-\sum_{i=q}^{P-1}\rho_{p-1,i}\cos\left(\xi_{p-1,i}+\varphi_{k}-i\cdot 2\pi \lambda^{-1}\psi_{x,k}\right)}{h_{c}^{\left(p\right)}},\;q=1,\ldots ,P-1$$

(27) $$\alpha_{s,q}^{\left(p\right)}=\frac{h_{s}^{\left(p\right)}-\sum_{i=q}^{P-1}\rho_{p-1,i}\sin\left(\xi_{p-1,i}+\varphi_{k}-i\cdot 2\pi \lambda^{-1}\psi_{x,k}\right)}{h_{s}^{\left(p\right)}},\;q=1,\ldots ,P-1$$

(28) $$\beta_{c,q}^{\left(p\right)}=\frac{h_{c}^{\left(p\right)}-\sum_{i=P-q}^{P-1}\rho_{p-1,i}\cos\left(\xi_{p-1,i}+\varphi_{k}+i\cdot 2\pi \lambda^{-1}\psi_{x,k}\right)}{h_{c}^{\left(p\right)}},\;q=1,\ldots ,P-1$$

(29) $$\beta_{s,q}^{\left(p\right)}=\frac{h_{s}^{\left(p\right)}-\sum_{i=P-q}^{P-1}\rho_{p-1,i}\sin\left(\xi_{p-1,i}+\varphi_{k}+i\cdot 2\pi \lambda^{-1}\psi_{x,k}\right)}{h_{s}^{\left(p\right)}},\;q=1,\ldots ,P-1$$

Similarly, the real-valued virtual steering vectors of $A_{I}$ can be defined as

(30) $$\begin{array}{c} v_{I}(\theta_{k},\phi_{k},\varphi_{k},C)=\sum_{p=1}^{P}\left(J_{p}a_{y,c}(\psi_{y,k})\right)⊗\left(\Gamma_{s,p}(\psi_{x,k})a_{x,c}(\psi_{x,k})\right)-\sum_{p=1}^{P}\left(J_{p}a_{y,s}(\psi_{y,k})\right)⊗\left(\Gamma_{s,p}(\psi_{x,k})a_{x,s}(\psi_{x,k})\right)\\ +\sum_{p=1}^{P}\left(J_{p}a_{y,c}(\psi_{y,k})\right)⊗\left(\Gamma_{c,p}(\psi_{x,k})a_{x,s}(\psi_{x,k})\right)+\sum_{p=1}^{P}\left(J_{p}a_{y,s}(\psi_{y,k})\right)⊗\left(\Gamma_{c,p}(\psi_{x,k})a_{x,c}(\psi_{x,k})\right) \end{array}$$

Based on the above real-valued signal model, we can concatenate the real part and the imaginary part to generate an augmented data vector in the real-valued domain.

(31) $$y\left(t\right)=\left[\begin{array}{c} x_{R}^{}(t)\\ x_{I}^{}(t) \end{array}\right]=\left[\begin{array}{c} A_{R}^{}\\ A_{I}^{} \end{array}\right]s_{0}^{}(t)+\left[\begin{array}{c} n_{R}^{}(t)\\ n_{I}^{}(t) \end{array}\right]=A_{E}^{}s_{0}^{}(t)+n_{E}^{}(t)$$

where $A_{E}^{}=[a_{E}^{}(\theta_{1}^{},\phi_{1}^{},\varphi_{1}^{},C),\cdots ,a_{E}^{}(\theta_{K}^{},\phi_{K}^{},\varphi_{K}^{},C)]$ is the augmented manifold matrix, and the corresponding real-valued steering vector of the *k*-th source is defined as

(32) $$a_{E}^{}(\theta_{k}^{},\phi_{k}^{},\varphi_{k}^{},C)=\left[\begin{array}{c} v_{R}^{}(\theta_{k}^{},\phi_{k}^{},\varphi_{k}^{},C)\\ v_{I}^{}(\theta_{k}^{},\phi_{k}^{},\varphi_{k}^{},C) \end{array}\right]$$

Therefore, the covariance matrix of the augmented real-valued data matrix **y**(*t*) is of the following form

(33) $$R_{y}=E\left\{y(t)y^{T}(t)\right\}=A_{E}R_{S_{0}}A_{E}^{T}+\sigma_{n}^{2}I_{2M}$$

where $R_{S_{0}}^{}=E\text{\{}s_{0}^{}(t)s_{0}^{T}(t)\text{\}}$, $I_{2M}$ denotes a 2*M* × 2*M* identity matrix. In practical applications, $R_{y}$ can be approximated from the snapshots generated at *L* distinct time instants

(34) $${\hat{R}}_{y}=\frac{1}{L}\sum_{t=1}^{L}\left\{y(t)y^{T}(t)\right\}$$

Through the eigenvalue decomposition of $R_{y}^{}$, we can obtain that

(35) $$R_{y}^{}=E_{s}^{}\Lambda_{s}^{}E_{s}^{H}+\sigma_{n}^{2}E_{n}^{}E_{n}^{H}$$

Herein, $\Lambda_{s}^{}$ is a diagonal matrix comprised of the largest *K* eigenvalues of $R_{y}^{}$. $E_{s}^{}$ is the signal subspace matrix which contains the eigenvectors corresponding to the largest *K* eigenvalues. $E_{n}^{}$ is the noise subspace matrix, which is composed of the remaining 2*M*-*K* eigenvectors of $R_{y}^{}$.

Based on the subspace principle that the noise subspace is orthogonal to the signal subspace, we can construct the following MUSIC spectrum function for estimating the azimuth and elevation angles of the sources.

(36) $$P(\theta ,\phi ,\varphi ,C)=a_{E}^{T}(\theta ,\phi ,\varphi ,C)E_{n}E_{n}^{T}a_{E}(\theta ,\phi ,\varphi ,C)$$

Unfortunately, this multidimensional spectrum function presented above requires a (*P* + 2)-dimension joint spectral search for 2D DOAs, MCCs, and noncircular phases, which is computationally prohibitive. Therefore, to decrease the complexity of the algorithm, we first factorize the augmented steering vector $a_{E}^{}(\theta_{k}^{},\phi_{k}^{},\varphi_{k}^{},C)$ with respect to the *y*-axis direction-cosine and present a novel real-valued RARE estimator in the next subsection, which only requires a simple 1D spectral search procedure.

### 3.2. First Stage Rank Reduction DOA Estimation

In this stage, the real-valued steering vector $a_{E}^{}$ in Equation (32) is first factorized with respect to the *y*-axis direction-cosine and other nuisance parameters. From Equations (17) and (30), the mutual coupling effect in the two virtual steering vectors can be deemed as angle-dependent gain and phase perturbations. However, it is clear that the central elements on the main diagonal of both $\Gamma_{c,p}(\psi_{x})$ and $\Gamma_{s,p}(\psi_{x})$ comprise a vector of all ones, which implies that the middle subarray can be handled as an unperturbed one. Therefore, we can obtain the following equations

(37) $$\Gamma_{c,p}(\psi_{x})a_{x,c}(\psi_{x})=T_{x,c}(\psi_{x})\alpha_{x,c}^{\left(p\right)}$$

(38) $$\Gamma_{c,p}(\psi_{x})a_{x,s}(\psi_{x})=T_{x,s}(\psi_{x})\alpha_{x,c}^{\left(p\right)}$$

(39) $$\Gamma_{s,p}(\psi_{x})a_{x,s}(\psi_{x})=T_{x,s}(\psi_{x})\alpha_{x,s}^{\left(p\right)}$$

(40) $$\Gamma_{s,p}(\psi_{x})a_{x,c}(\psi_{x})=T_{x,c}(\psi_{x})\alpha_{x,s}^{\left(p\right)}$$

where $T_{x,c}(\psi_{x})$ and $T_{x,s}(\psi_{x})$ are $M_{x}\times (2P-1)$ real matrices, and $\alpha_{x,c}^{\left(p\right)}$ and $\alpha_{x,s}^{\left(p\right)}$ are (2P−1)×1 real vectors, which can be expressed as

(41) $$T_{x,c}(\psi_{x})=\left[\begin{array}{cccccc} 1 & & & & & \\ & \cos(\psi_{x}) & & & & 0\\ & & \ddots & & & \\ & & & \cos((P-1)\psi_{x}) & & \\ & & & \vdots & & \\ & & & \cos((M_{x}-P)\psi_{x}) & & \\ & 0 & & & \ddots & \\ & & & & & \cos((M_{x}-1)\psi_{x}) \end{array}\right]$$

(42) $$T_{x,s}(\psi_{x})=\left[\begin{array}{cccccc} 0 & & & & & \\ & \sin(\psi_{x}) & & & & 0\\ & & \ddots & & & \\ & & & \sin((P-1)\psi_{x}) & & \\ & & & \vdots & & \\ & & & \sin((M_{x}-P)\psi_{x}) & & \\ & 0 & & & \ddots & \\ & & & & & \sin((M_{x}-1)\psi_{x}) \end{array}\right]$$

(43) $$\alpha_{x,c}^{\left(p\right)}=h_{c}^{\left(p\right)}{\left[\alpha_{c,1}^{\left(p\right)},\cdots ,\alpha_{c,P-1}^{\left(p\right)},1,\beta_{c,1}^{\left(p\right)},\cdots ,\beta_{c,P-1}^{\left(p\right)}\right]}^{T}$$

(44) $$\alpha_{x,s}^{\left(p\right)}=h_{s}^{\left(p\right)}{\left[\alpha_{s,1}^{\left(p\right)},\cdots ,\alpha_{s,P-1}^{\left(p\right)},1,\beta_{s,1}^{\left(p\right)},\cdots ,\beta_{s,P-1}^{\left(p\right)}\right]}^{T}$$

Similarly, for the *y*-axis direction-cosine related steering vector, we have that

(45) $$J_{p}a_{y,c}(\psi_{y})=T_{y,c}(\psi_{y})\alpha_{y,c}^{\left(p\right)}-T_{y,s}(\psi_{y})\alpha_{y,s}^{\left(p\right)}$$

(46) $$J_{p}a_{y,s}(\psi_{y})=T_{y,s}(\psi_{y})\alpha_{y,c}^{\left(p\right)}+T_{y,c}(\psi_{y})\alpha_{y,s}^{\left(p\right)}$$

where $T_{y,c}(\psi_{y})$ and $T_{y,s}(\psi_{y})$ are $M_{y}\times (2P-1)$ real matrices, and $\alpha_{y,c}^{\left(p\right)}$ and $\alpha_{y,s}^{\left(p\right)}$ are (2P−1)×1 real vectors, which can be expressed as

(47) $$T_{y,c}(\psi_{y})=\left[\begin{array}{cccccc} 1 & & & & & \\ & \cos(\psi_{y}) & & & & 0\\ & & \ddots & & & \\ & & & \cos((P-1)\psi_{y}) & & \\ & & & \vdots & & \\ & & & \cos((M_{y}-P)\psi_{y}) & & \\ & 0 & & & \ddots & \\ & & & & & \cos((M_{y}-1)\psi_{y}) \end{array}\right]$$

(48) $$T_{y,s}(\psi_{y})=\left[\begin{array}{cccccc} 0 & & & & & \\ & \sin(\psi_{y}) & & & & 0\\ & & \ddots & & & \\ & & & \sin((P-1)\psi_{y}) & & \\ & & & \vdots & & \\ & & & \sin((M_{y}-P)\psi_{y}) & & \\ & 0 & & & \ddots & \\ & & & & & \sin((M_{y}-1)\psi_{y}) \end{array}\right]$$

For simplicity, we also denote $T_{y,c}(\psi_{y})$ as $T_{y,c}$ in the following derivation. Based on the relationships derived above, we can factorize the virtual steering vectors $v_{R}$ and $v_{I}$ in Equations (17) and (30) as

(49) $$\begin{array}{l} v_{R}(\theta ,\phi ,\varphi ,C)=\\ \;(T_{y,c}\alpha_{y,c})⊗(T_{x,c}\alpha_{x,c})-(T_{y,c}\alpha_{y,c})⊗(T_{x,s}\alpha_{x,s})-(T_{y,s}\alpha_{y,s})⊗(T_{x,c}\alpha_{x,c})+(T_{y,s}\alpha_{y,s})⊗(T_{x,s}\alpha_{x,s})\\ \;-(T_{y,s}\alpha_{y,c})⊗(T_{x,s}\alpha_{x,c})-(T_{y,s}\alpha_{y,c})⊗(T_{x,c}\alpha_{x,s})-(T_{y,c}\alpha_{y,s})⊗(T_{x,s}\alpha_{x,c})-(T_{y,c}\alpha_{y,s})⊗(T_{x,c}\alpha_{x,s}) \end{array}$$

(50) $$\begin{array}{l} v_{I}(\theta ,\phi ,\varphi ,C)=\\ \;(T_{y,c}\alpha_{y,c})⊗(T_{x,c}\alpha_{x,s})+(T_{y,c}\alpha_{y,c})⊗(T_{x,s}\alpha_{x,c})-(T_{y,s}\alpha_{y,s})⊗(T_{x,c}\alpha_{x,s})-(T_{y,s}\alpha_{y,s})⊗(T_{x,s}\alpha_{x,c})\\ \;-(T_{y,s}\alpha_{y,c})⊗(T_{x,s}\alpha_{x,s})+(T_{y,s}\alpha_{y,c})⊗(T_{x,c}\alpha_{x,c})-(T_{y,c}\alpha_{y,s})⊗(T_{x,s}\alpha_{x,s})+(T_{y,c}\alpha_{y,s})⊗(T_{x,c}\alpha_{x,c}) \end{array}$$

where $\alpha_{x,c}$, $\alpha_{x,s}$, $\alpha_{y,c}$, and $\alpha_{y,s}$ are defined as

(51) $$\alpha_{x,c}=\sum_{p=1}^{P}\alpha_{x,c}^{\left(p\right)},\;\alpha_{x,s}=\sum_{p=1}^{P}\alpha_{x,s}^{\left(p\right)}$$

(52) $$\alpha_{y,c}=\sum_{p=1}^{P}\alpha_{y,c}^{\left(p\right)},\;\alpha_{y,s}=\sum_{p=1}^{P}\alpha_{y,s}^{\left(p\right)}$$

Therefore, utilizing the Kronecker product property (AB)⊗(CD)=(A⊗C)(B⊗D), we can write the virtual steering vector $a_{E}^{}$ as the following expression

(53) $$\begin{array}{ll} a_{E}(\psi_{x},\psi_{y},\varphi ,C) & =\left[\begin{array}{c} v_{R}\\ v_{I} \end{array}\right]\\ & =\left[\begin{array}{cc} \left(T_{y,c}⊗I_{x}\right)\left(I_{y}⊗T_{x,c}\right)-\left(T_{y,s}⊗I_{x}\right)\left(I_{y}⊗T_{x,s}\right) & -\left(T_{y,c}⊗I_{x}\right)\left(I_{y}⊗T_{x,s}\right)-\left(T_{y,s}⊗I_{x}\right)\left(I_{y}⊗T_{x,c}\right)\\ \left(T_{y,c}⊗I_{x}\right)\left(I_{y}⊗T_{x,s}\right)+\left(T_{y,s}⊗I_{x}\right)\left(I_{y}⊗T_{x,c}\right) & \left(T_{y,c}⊗I_{x}\right)\left(I_{y}⊗T_{x,c}\right)-\left(T_{y,s}⊗I_{x}\right)\left(I_{y}⊗T_{x,s}\right) \end{array}\right]\left[\begin{array}{c} \alpha_{y,c}⊗\alpha_{x,c}-\alpha_{y,s}⊗\alpha_{x,c}\\ \alpha_{y,c}⊗\alpha_{x,s}-\alpha_{y,s}⊗\alpha_{x,c} \end{array}\right] \end{array}$$

where **I***_x_* is a *M_x_* × *M_x_* identity matrix and **I***_y_* is a (2*P* − 1) × (2*P* − 1) identity matrix. By factorizing the first matrix, we can reorganize $a_{E}^{}$ in the following form

(54) $$\begin{array}{ll} a_{E}(\psi_{x},\psi_{y},\varphi ,C) & =\left[\begin{array}{c} v_{R}\\ v_{I} \end{array}\right]\\ & \;=\underset{T_{1}(\psi_{y})}{\underset{︸}{\left[\begin{array}{cc} T_{y,c}⊗I_{x} & -T_{y,s}⊗I_{x}\\ T_{y,s}⊗I_{x} & T_{y,c}⊗I_{x} \end{array}\right]}}\underset{\alpha_{1}(\psi_{x},\psi_{y},\varphi ,C)}{\underset{︸}{\left[\begin{array}{cc} I_{y}⊗T_{x,c} & -I_{y}⊗T_{x,s}\\ I_{y}⊗T_{x,s} & I_{y}⊗T_{x,c} \end{array}\right]\left[\begin{array}{c} \alpha_{y,c}⊗\alpha_{x,c}-\alpha_{y,s}⊗\alpha_{x,c}\\ \alpha_{y,c}⊗\alpha_{x,s}-\alpha_{y,s}⊗\alpha_{x,c} \end{array}\right]}} \end{array}$$

where $T_{1}(\psi_{y})$ is a $2M_{x}M_{y}\times 2M_{x}(2P-1)$ matrix which only depends on the *y*-axis direction-cosine, and $\alpha_{1}(\psi_{x},\psi_{y},\varphi ,C)$ is a $2M_{x}(2P-1)\times 1$ vector which depends on the *x*-axis and *y*-axis direction-cosines, MCCs, and noncircular phases.

Substituting the above equation into Equation (36), we have

(55) $$\alpha_{1}^{T}(\psi_{x},\psi_{y},\varphi ,C)Q_{1}(\psi_{y})\alpha_{1}(\psi_{x},\psi_{y},\varphi ,C)=0$$

where $Q_{1}(\psi_{y})$ is defined as

(56) $$Q_{1}(\psi_{y})=T_{1}^{T}(\psi_{y})E_{n}E_{n}^{T}T_{1}(\psi_{y})$$

It is obvious that $Q_{1}(\psi_{y})$ is a $2M_{x}(2P-1)\times 2M_{x}(2P-1)$ real matrix, $T_{1}(\psi_{y})$ is a $2M_{x}M_{y}\times 2M_{x}(2P-1)$ real matrix, and $E_{n}$ is the $2M_{x}M_{y}\times (2M_{x}M_{y}-K)$ real-valued noise subspace matrix. Accordingly, if $2M_{x}M_{y}-K\geq 2M_{x}(2P-1)$, $Q_{1}(\psi_{y})$ will generally be of full column rank. Note that $\alpha_{1}$ is presumed to be a non-zero vector, thus Equation (55) holds only when $Q_{1}(\psi_{y})$ rank drops, which implies that $Q_{1}(\psi_{y})$ becomes a rank-deficient matrix when $\psi_{y}=\psi_{y,k},\;k=1,\cdots ,K$. Therefore, based on the principle of RARE, the *y*-axis direction-cosines can be estimated from the highest peaks of the following 1D spectral search function:

(57) $$P_{1}(\psi_{y})=\frac{1}{\det\left[T_{1}^{T}(\psi_{y})E_{n}E_{n}^{T}T_{1}(\psi_{y})\right]}$$

Since the *y*-axis direction-cosine $\psi_{y}$ has been decoupled with other nuisance parameters in this stage, we can simplify the previous multidimensional optimization problem in Equation (36) into a 1D spectral search problem, which is computationally more efficient. Additionally, the operations involved in this stage are all real-valued, which means the computational load can be reduced by a factor of four compared with its complex-valued counterparts.

Our next objective is to estimate the values of *x*-axis direction-cosines and correctly pair them with the corresponding *y*-axis direction-cosines. Therefore, in the following subsection, we will present another RARE function which can generate the estimation of *x*-axis direction-cosines $\psi_{x,k},k=1,\cdots ,K$ without any additional matching procedures.

### 3.3. Second Stage Rank Reduction DOA Estimation

In the second stage, the real-valued steering vector $a_{E}^{}$ in Equation (32) is factorized in another form with respect to the *x*-axis direction-cosine and other nuisance parameters. Since the *y*-axis direction-cosine $\psi_{y}$ has already been obtained in the previous stage, we can calculate the values of $\sum_{p=1}^{P}\left(J_{p}a_{y,c}(\psi_{y,k})\right)$ and $\sum_{p=1}^{P}\left(J_{p}a_{y,s}(\psi_{y,k})\right)$. Also we denote that

(58) $$\beta_{y,c}=\sum_{p=1}^{P}\left(J_{p}a_{y,c}(\psi_{y,k})\right),\;\beta_{y,s}=\sum_{p=1}^{P}\left(J_{p}a_{y,s}(\psi_{y,k})\right)$$

Therefore, the virtual steering vectors $v_{R}$ and $v_{I}$ in Equations (17) and (30) can be also factorized as

(59) $$v_{R}(\theta_{k},\phi_{k},\varphi_{k},C)=\beta_{y,c}⊗\left[T_{x,c}\alpha_{x,c}^{}-T_{x,s}\alpha_{x,s}^{}\right]-\beta_{y,s}⊗\left[T_{x,s}\alpha_{x,c}^{}+T_{x,c}\alpha_{x,s}^{}\right]$$

(60) $$v_{I}(\theta_{k},\phi_{k},\varphi_{k},C)=\beta_{y,c}⊗\left[T_{x,c}\alpha_{x,s}^{}+T_{x,s}\alpha_{x,c}^{}\right]-\beta_{y,s}⊗\left[T_{x,s}\alpha_{x,s}^{}-T_{x,c}\alpha_{x,c}^{}\right]$$

Utilizing the Kronecker product property β⊗(T⋅α)=(β⊗T)⋅α, we have the following expression

(61) $$v_{R}(\theta_{k},\phi_{k},\varphi_{k},C)=\left(\beta_{y,c}⊗T_{x,c}\right)\alpha_{x,c}^{}-\left(\beta_{y,c}⊗T_{x,s}\right)\alpha_{x,s}^{}-\left(\beta_{y,s}⊗T_{x,s}\right)\alpha_{x,c}^{}-\left(\beta_{y,s}⊗T_{x,c}\right)\alpha_{x,s}^{}$$

(62) $$v_{I}(\theta_{k},\phi_{k},\varphi_{k},C)=\left(\beta_{y,c}⊗T_{x,c}\right)\alpha_{x,s}^{}+\left(\beta_{y,c}⊗T_{x,s}\right)\alpha_{x,c}^{}-\left(\beta_{y,s}⊗T_{x,s}\right)\alpha_{x,s}^{}+\left(\beta_{y,s}⊗T_{x,c}\right)\alpha_{x,c}^{}$$

where $I_{\beta }$ is a M_y_ × M_y_ identity matrix. Consequently, the extended real-valued steering vector $a_{E}$ can be written in the following form

(63) $$a_{E}(\psi_{x},\psi_{y},\varphi ,C)=\left[\begin{array}{c} v_{R}\\ v_{I} \end{array}\right]=\underset{T_{2}(\psi_{x})}{\underset{︸}{\left[\begin{array}{cc} \beta_{y,c}⊗T_{x,c}-\beta_{y,s}⊗T_{x,s} & -\beta_{y,c}⊗T_{x,s}-\beta_{y,s}⊗T_{x,c}\\ \beta_{y,c}⊗T_{x,s}+\beta_{y,s}⊗T_{x,c} & \beta_{y,c}⊗T_{x,c}-\beta_{y,s}⊗T_{x,s} \end{array}\right]}}\underset{\alpha_{2}(\psi_{x},\varphi ,C)}{\underset{︸}{\left[\begin{array}{c} \alpha_{x,c}\\ \alpha_{x,s} \end{array}\right]}}$$

where $T_{2}(\psi_{x})$ is a $2M_{x}M_{y}\times 2(2P-1)$ matrix which only depends on the *x*-axis direction-cosine, and $\alpha_{2}(\psi_{x},\varphi ,C)$ is a 2(2P−1) vector which depends on the *x*-axis direction-cosines, MCCs, and noncircular phases.

Again, according to the RARE principle, we substitute the above equation into Equation (36)

(64) $$\alpha_{2}^{T}(\psi_{x},\varphi ,C)Q_{2}(\psi_{x})\alpha_{2}(\psi_{x},\varphi ,C)=0$$

where $Q_{2}(\psi_{x})$ is defined as

(65) $$Q_{2}(\psi_{x})=T_{2}^{T}(\psi_{x})E_{n}E_{n}^{T}T_{2}(\psi_{x}).$$

Note that $Q_{2}(\psi_{x})\in {\mathbb{R}}^{2(2P-1)\times 2(2P-1)}$ and $T_{2}(\psi_{x})\in {\mathbb{R}}^{2M_{x}M_{y}\times 2(2P-1)}$. If $2M_{x}M_{y}-K\geq 2(2P-1)$, then $Q_{2}(\psi_{x})$ is of full column rank in general. As both $\alpha_{x,c}$ and $\alpha_{x,s}$ are supposed to be non-zero vectors, $\alpha_{2}$ must be non-zero as well. One can deduce that (64) holds only if the rank of $Q_{2}(\psi_{x})$ drops, that is, rank deficiency occurs in $Q_{2}(\psi_{x})$ provided that $\psi_{x}=\psi_{x,k},\;k=1,\cdots ,K$. By the principle of RARE, the *x*-axis direction-cosines can be obtained by searching for the highest peaks of the following spatial spectrum function:

(66) $$P_{2}(\psi_{x})=\frac{1}{\det\left[T_{2}^{T}(\psi_{x})E_{n}E_{n}^{T}T_{2}(\psi_{x})\right]}.$$

Since the *x*-axis direction-cosine $\psi_{x}$ has been decoupled with other nuisance parameters in the second stage, the multidimensional optimization problem can be reduced to a computationally efficient 1D spectral searching problem. Moreover, the computations involved are all real-valued, which means the computational load can be reduced by a factor of four compared with its complex-valued counterparts. Also, due to the orthogonality between the signal subspace and noise subspace, the 2D DOA parameters are automatically paired without any additional matching procedures.

Therefore, the 2D DOA estimates can be obtained from the following equations:

(67) $${\hat{\theta }}_{k}=\mathrm{arc}\cot\left(\left(\frac{\lambda {\hat{\psi }}_{x}}{2\pi d_{x}}\right)/\left(\frac{\lambda {\hat{\psi }}_{y}}{2\pi d_{y}}\right)\right)$$

(68) $${\hat{\phi }}_{k}=\arcsin\left(\sqrt{{\left(\frac{\lambda {\hat{\psi }}_{x}}{2\pi d_{x}}\right)}^{2}+{\left(\frac{\lambda {\hat{\psi }}_{y}}{2\pi d_{y}}\right)}^{2}}\right)$$

### 3.4. Procedures of the Proposed Algorithm

The steps of the proposed algorithm can be summarized as follows.

(1) Collect the real and imaginary parts of **x**(*t*) based on Equation (12);

(2) construct the augmented data vector via Equation (31);

(3) calculate the covariance matrix of **y**(*t*) by Equation (34);

(4) perform the eigenvalue decomposition of ${\hat{R}}_{y}^{}$, and extract the noise subspace matrix ${\hat{E}}_{n}^{}$;

(5) formulate the matrix $T_{1}(\psi_{y})$ for each *y*-axis direction-cosine searching grid according to Equation (54);

(6) find the estimation of *y*-axis direction-cosines $\psi_{y,k},k=1,\cdots ,K$ through the 1D spectral search in Equation (57);

(7) compute the values of $\beta_{y,c}$, $\beta_{y,s}$ and $T_{2}(\psi_{x})$ for each *x*-axis direction-cosines estimates based on Equations (58) and (63);

(8) obtain the estimates of *x*-axis direction-cosines $\psi_{x,k},k=1,\cdots ,K$ according to Equation (66);

(9) calculate the DOA estimates of the azimuth and elevation angles according to Equations (67) and (68).

## 4. Discussion

In this section, we compare the performance of the proposed algorithm with respect to two state-of-art 2D DOA estimation algorithms considering mutual coupling effects: the auxiliary sensor-based method (AUX) in [39] and the 2D rank reduction-based method (2D-RARE) in [40].

### 4.1. Computational Complexity

In this subsection, the computational complexity of the proposed algorithm is evaluated with respect to AUX and 2D-RARE. Note that the computational complexity is investigated in terms of the major multiplications in constructing the statistical matrices, EVDs, and spectral searches. Suppose that an *M_x_* × *M_y_* URA is utilized and the grid size for spectral searching is Δθ. From [29], it can be observed that AUX constructs a (*M_x_* − 2*P* + 2)(*M_y_* − 2*P* + 2) × (*M_x_* − 2*P* + 2)(*M_y_* − 2*P* + 2) covariance matrix, takes the EVD of this matrix, and performs a 2D spectral search for DOA estimation, which require a computational complexity of $O(4{\left(M_{x}-2P+2\right)}^{2}{\left(M_{y}-2P+2\right)}^{2}L)$, $O(4{\left(M_{x}-2P+2\right)}^{3}{\left(M_{y}-2P+2\right)}^{3}/3)$, and $O(4{\left(M_{x}-2P+2\right)}^{2}{\left(M_{y}-2P+2\right)}^{2}(360{}^{\circ}/\Delta \theta )(90{}^{\circ}/\Delta \theta ))$, respectively. 2D-RARE constructs a *M_x_M_y_* × *M_x_M_y_* covariance matrix, performs the EVD, calculates the rank reduction testing matrix, and performs a 2D RARE spectral search, which require a computational complexity of $O(4{\left(M_{x}M_{y}\right)}^{2}L)$, $O(4{\left(M_{x}M_{y}\right)}^{3}/3)$, and $O\left(\left(360{}^{\circ}/\Delta \theta )(90{}^{\circ}/\Delta \theta ))(M_{x}M_{y}(M_{x}M_{y}-K){\left(2P-1\right)}^{2}+(M_{x}M_{y}-K){\left(2P-1\right)}^{4}+{\left(M_{x}M_{y}\right)}^{3}\right)\right)$, respectively [30]. For the proposed algorithm, the establishment of the 2*M_x_M_y_* × 2*M_x_M_y_* real-valued statistical matrix takes $O(4{\left(M_{x}M_{y}\right)}^{2}L)$ operations, the EVD of this matrix requires $O(8{\left(M_{x}M_{y}\right)}^{3}/3)$ multiplications, the first stage of the 1D RARE spectral search takes $O\left((360{}^{\circ}/\Delta \theta ){\left(2M_{x}(2P-1)\right)}^{3}\right)$, and the second stage of the 1D RARE spectral search has a complexity of $O\left(K\cdot (90{}^{\circ}/\Delta \theta ){\left(4P-2\right)}^{3}\right)$. It is worth noting that the calculations involved in the proposed algorithm are in the real-valued domain. Thus, with the same matrix dimensions, the computational load can be effectively reduced by a factor of four.

### 4.2. Maximum Number of Resolvable Sources

In this subsection, we compare the maximum number of resolvable sources for the three algorithms. Note that AUX only utilizes the (*M_x_* − 2*P* + 2)(*M_y_* − 2*P* + 2) sensors in the middle of the URA. As a result of the subspace identification technique, at least one eigenvector is required for spanning the noise subspace. Therefore, AUX is capable of resolving no more than (*M_x_* − 2*P* + 2)(*M_y_* − 2*P* + 2) − 1 sources. Similarly, 2D-RARE requires that (2*P* − 1)^2^ ≤ *M_x_M_y_*-*K*. Thus, it can also resolve *M_x_M_y_* − (2*P* − 1)^2^ sources at most. For the proposed method, it requires $2M_{x}M_{y}-K\geq 2M_{x}(2P-1)$ and $2M_{x}M_{y}-K\geq 2(2P-1)$ in the two consequent RARE estimators, which means the maximum number of resolvable sources for the proposed method is $2M_{x}M_{y}-2M_{x}(2P-1)$. Therefore, it can be concluded that the alleviation of computational loads is at the cost of DOF reduction, since the signal subspace has been decoupled.

### 4.3. Dependence of Mutual Coupling on Directions

In this subsection, we discuss the dependence of antenna mutual coupling effects on the angles of signals. According to the latest work by B. Friedlander [28], the mutual coupling matrix is a direction dependent matrix, and not a constant matrix as is commonly assumed. In this work, the mutual coupling matrix of the URA is assumed to be direction-independent with a block banded Toeplitz structure, which has been simplified compared with the analytic manifold [28]. Therefore, the DOA estimation performance may deteriorate due to the mismatch between the assumed model and the physical characteristics of the array antennas.

## 5. Simulation Results

In this section, we present several numerical simulations to analyze the DOA estimation performance of the proposed algorithm with respect to 2D-MUSIC, AUX, and 2D-RARE. In the following simulations, a 10 × 10-elements URA is employed with the inter-element spacing being *d_x_* = *d_y_* =λ/2. The impinging sources are BPSK modulated with equal power and uncorrelated to each other, while the noise is amenable to the spatially white complex Gaussian distribution. The mutual coupling length is assumed to be P=2, and MCCs are $c_{0,1}=c_{1,0}=0.7527+j0.4854$, $c_{1,1}=0.2825+j0.2801$. The estimation performance is assessed in terms of spatial spectrum, root mean-square error (RMSE), and computational complexity. The RMSE is measured by 500 independent Monte Carlo runs:

(69) $$RMSE=\sqrt{\frac{1}{1000K}\sum_{n=1}^{500}\sum_{k=1}^{K}\left({\left(\theta_{k}-{\hat{\theta }}_{n,k}\right)}^{2}+{\left(\phi_{k}-{\hat{\phi }}_{n,k}\right)}^{2}\right)}$$

where $\theta_{k}$ is the DOA of the *k*-th source, and ${\hat{\theta }}_{n,k}$ stands for the estimate of  in the *n*th trial.

**Figure 2:**
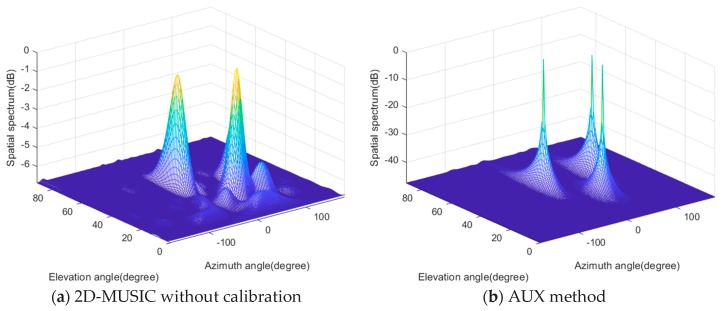

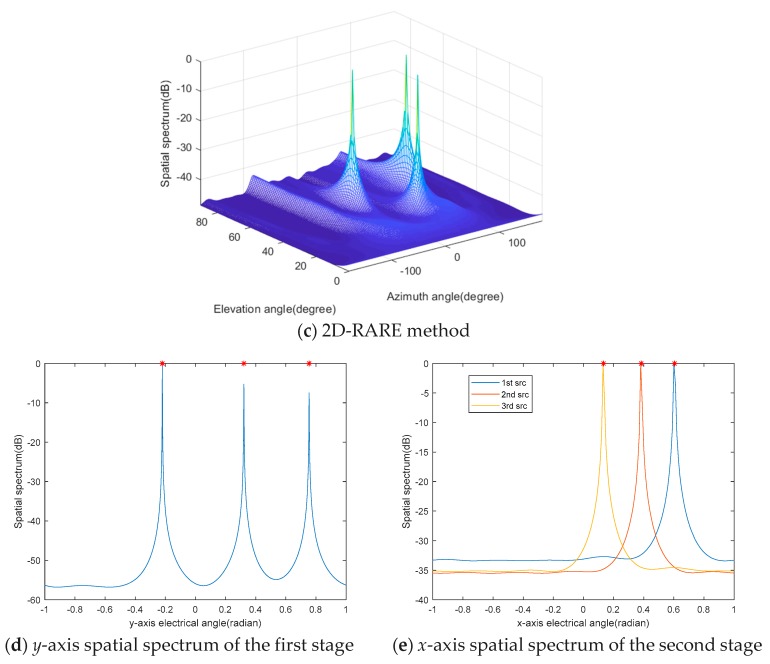
The spatial spectra of (**a**) Two-dimensional multiple signal classification (2D-MUSIC), (**b**) the auxiliary sensor-based method (AUX), (**c**) the 2D rank reduction-based method (2D-RARE) and (**d**) the first stage and (**e**) the second stage of the proposed method.

In the first case, we investigate the DOA spectra of the three algorithms. We consider that three incident rectilinear signals radiate from ($\theta_{1}=-20{}^{\circ},\phi_{1}=40{}^{\circ}$), ($\theta_{2}=40{}^{\circ},\phi_{2}=30{}^{\circ}$), and ($\theta_{3}=80{}^{\circ},\phi_{3}=50{}^{\circ}$), respectively. The corresponding *x*-axis direction-cosines are (0.604, 0.383, 0.133) in radians, and *y*-axis direction-cosines are (–0.220, 0.321, 0.754) in radians, respectively. The signal-to-noise ratio (SNR) is 10 dB, and the number of snapshots is fixed at 1000. In Figure 2d, the proposed algorithm generates three sharp peaks corresponding to the *y*-axis direction-cosines of the three BPSK sources in the first DOA estimation stage. In Figure 2e, the three spectra produce three sharp peaks at the desired locations of the *x*-axis direction-cosines, respectively. Note that the DOA estimates of in the two stages are automatically paired without any postprocessing. From Figure 2a, it is can be seen that 2D-MUSIC fails to distinguish all three sources, and its two resolvable spectra peaks are biased due to the mismatch caused by the mutual coupling effects. Although AUX and 2D-RARE can generate three peaks corresponding to the sources in the 2D DOA spectrum plain, the former sacrifices eight sensors (i.e., the auxiliary sensors) to make the remaining part of the URA coupling-free while the latter requires exhaustive 2D spectral searches to obtain the joint azimuth and elevation DOA estimates.

**Figure 3:**
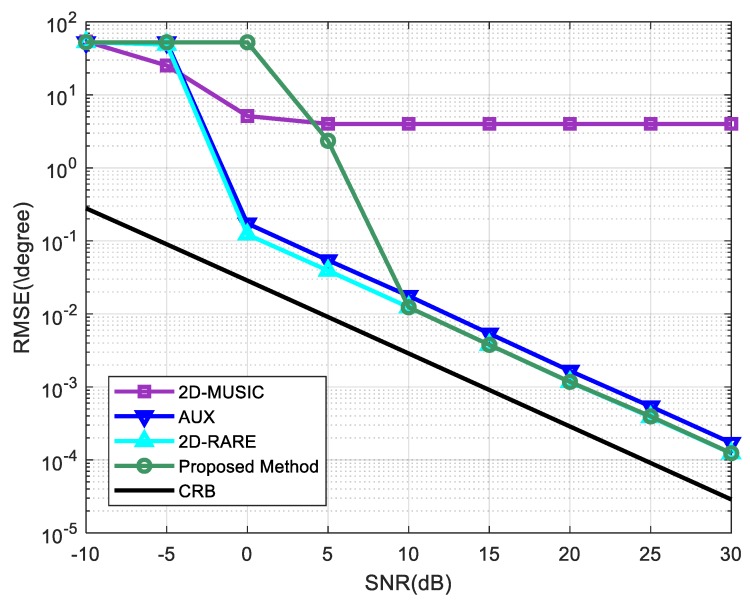
RMSEs of Root mean square errors (RMSEs) of direction-of-arrival (DOA) estimates versus signal-to-noise ratio (SNR). The number of snapshots is 1000.

In the second experiment, we studied the RMSE of the DOA estimates as a function of the SNR. The number of snapshots remained at 1000. Other simulation settings were the same as the first experiment. Figure 3 depicts the RMSE of the proposed method while the RMSEs of the other three solutions, 2D-MUSIC, AUX, 2D-RARE, and the corresponding CRB for noncircular source DOA estimation in the presence of mutual coupling is also calculated for comparison. It is clear that 2D-RARE performs the best among the four algorithms, overall AUX takes second place, our method provides better accuracies than it at 10 dB and above but is inferior to AUX at low SNRs, and 2D-MUSIC has the worst performance due to the low robustness to the unknown mutual coupling. Besides, the performance of the proposed method, 2D-RARE, and AUX bears down on the CRB asymptotically at high SNRs, while the RMSE of 2D-MUSIC is stabilized at approximately $4^{\circ }$ when the SNR is larger than 5dB.

**Figure 4:**
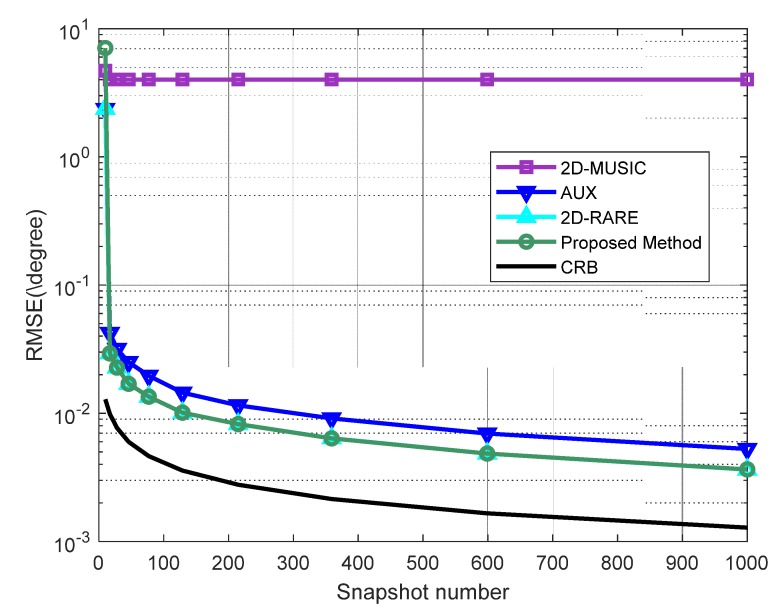
RMSEs of DOA estimates versus the number of snapshots. The SNR is 10 dB.

In the third experiment, we examine the variation of RMSE with the increase of the number of snapshots from 10 to 1000, where the SNR is set as 10dB. Figure 4 demonstrates that RMSEs of the proposed method, 2D-RARE, and AUX decline steadily as the snapshot size raises. This is because that the covariance and elliptic covariance matrices can be estimated more accurately from a larger number of observations. Additionally, the performance of our developed approach is almost the same as 2D-RARE and strictly superior to AUX, asymptotically approaching the corresponding CRB, while the RMSE of 2D-MUSIC saturates at $4^{\circ }$ as similar to the second scenario, which is the worst performance.

**Figure 5:**
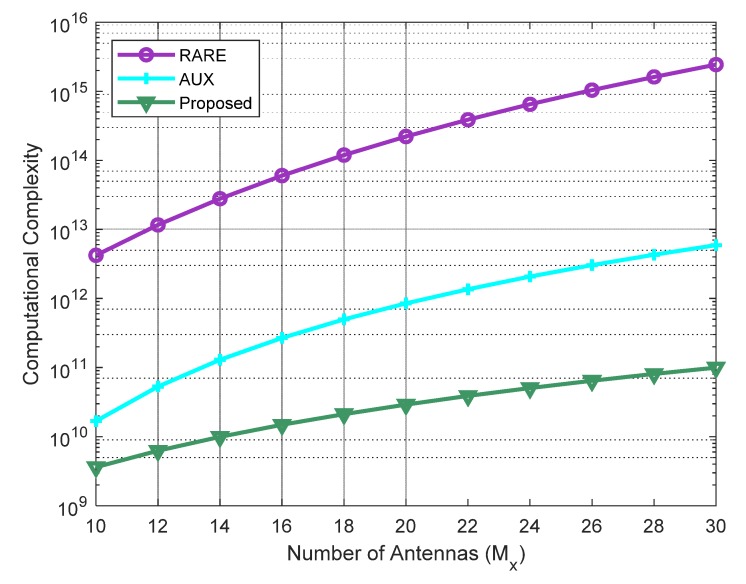
Computational complexity of the four methods versus number of antennas.

In the last experiment, the computational burdens of the three algorithms versus the variation of the number of sensors were compared. The searching step size for DOA estimation was set as 0.1°, and the number of snapshots was fixed at 200. Suppose the URA is square, i.e., $M_{x}=M_{y}$, and the number of elements ranges from 10 to 40. It can be observed from Figure 5 that our method is the most efficient compared with other two as it is only reliant upon a 1D spectral search, followed by AUX, and 2D-RARE requires the highest complexity.

## 6. Conclusions

In this paper, a 2D real-valued DOA estimator that is robust to unknown mutual coupling was developed for rectilinear sources. Taking advantage of the noncircularity in signals and the structured mutual coupling matrix revealed as block Toeplitz, a RARE estimator in real-value field was proposed to extract the angular information from the coupling coefficients. Compared with state-of-the-art approaches, our solution has satisfactory estimation performance with more efficient computations. A series of numerical simulations showed that the proposed method has its own advantages on estimation accuracy and computational complexity over previous algorithms with a favorable robustness to unknown mutual coupling. To apply the proposed algorithm to further applications, we may extend the proposed algorithm to considering the physical characteristics of the array antennas, such as direction-dependent mutual coupling and unstructured mutual coupling matrix in further studies.

## Figures and Tables

**Table 1:** List of notations.

Notations	Explanation
${\left(\cdot \right)}^{*}$	conjugate operator
${\left(\cdot \right)}^{T}$	transpose operator
${\left(\cdot \right)}^{H}$	conjugate transpose operator
Re{·}	real part of the operand
Im{·}	imaginary part of the operand
E{⋅}	expectation operator
vec(·)	vectorization operator
e	Hadamard product operator
⊗	Kronecker product operator
Tr{·}	trace of the operand
det(·)	determinant of the operand
**I** *_n_*	identity matrix of order *n*

## Appendix A

In the Appendix, we derive the unconditional or stochastic CRB of DOA estimation for noncircular sources under unknown mutual coupling effects. Since the signals are rectilinear (also called strictly second-order noncircular), $s_{0}(t)$ is zero-mean real-valued Gaussian distributed with covariance $E\left(s_{0}(t)s_{0}^{T}(t)\right)=R_{s}$. Furthermore, the model can be parameterized by the following real-valued unknown parameter vector $\Theta =[\theta^{T},\phi^{T},\varphi^{T},c_{R}^{T},c_{I}^{T},\rho^{T},\sigma_{n}^{2}]$, where $\theta ={\left[\theta_{1},\cdots ,\theta_{K}\right]}^{T}$ denotes the azimuth angle vector, $\phi ={\left[\phi_{1},\cdots ,\phi_{K}\right]}^{T}$ represents the elevation angle vector, $\varphi ={\left[\varphi_{1},\cdots ,\varphi_{K}\right]}^{T}$ contains the noncircular phases, $c_{R}$ and $c_{I}$ are the real and imaginary parts of the mutual coupling coefficients vector, and $\rho ={\left[\rho_{1},\cdots ,\rho_{K}\right]}^{T}$ is the *K* × 1 vector made from ${\left[R_{s}\right]}_{i,i}$, 1≤ *i* ≤*K*. Based on the results in [42], the Fisher information matrix (FIM) corresponding to the parameter vector Θ can be written as:

(A1) $$F_{m,n}=L\cdot \mathrm{Tr}\left(\frac{\partial R_{\bar{x}}}{\partial \Theta_{m}}R_{\bar{x}}^{-1}\frac{\partial R_{\bar{x}}}{\partial \Theta_{n}}R_{\bar{x}}^{-1}\right)$$

where $R_{\bar{x}}$ is the extended covariance matrix corresponding to the augmented signal $\bar{x}(t)={\left[x_{}^{T}(t),x_{}^{H}(t)\right]}^{T}$, which is formed as:

(A2) $$R_{\bar{x}}=E\{\bar{x}(t){\bar{x}}^{H}(t)\}=\bar{A}R_{s}{\bar{A}}^{H}+\sigma_{n}^{2}I$$

where $\bar{A}=\left[\begin{array}{c} CA\Phi \\ C^{*}A^{*}\Phi^{*} \end{array}\right]=\left[{\bar{a}}_{1},\cdots ,{\bar{a}}_{K}\right]$, with ${\bar{a}}_{k}=\left[\begin{array}{c} e^{j\varphi_{k}}Ca(\theta_{k},\phi_{k})\\ e^{-j\varphi_{k}}C^{*}a^{*}(\theta_{k},\phi_{k}) \end{array}\right]$.

The FIM for the unknown parameters and their cross terms can be readily derived following the steps in [43]. Since **F** is a symmetric matrix, we only need to find the upper triangular parts of **F**, and the block matrices in **F** can be obtained as:

(A3) Fθθ=2L⋅Re{(RsA¯HRx¯−1A¯˙θ)⊙(RsA¯HRx¯−1A¯˙θ)T+(RsA¯HRx¯−1A¯Rs)⊙(A¯˙θHRx¯−1A¯˙θ)T}

(A4) Fϕϕ=2L⋅Re{(RsA¯HRx¯−1A¯˙ϕ)⊙(RsA¯HRx¯−1A¯˙ϕ)T+(RsA¯HRx¯−1A¯Rs)⊙(A¯˙ϕHRx¯−1A¯˙ϕ)T}

(A5) Fφφ=2L⋅Re{(RSA¯HRx¯−1A¯˙φ)⊙(RSA¯HRx¯−1A¯˙φ)T+(RSA¯HRx¯−1A¯RS)⊙(A¯˙φHRx¯−1A¯˙φ)T}

(A6) FcR,mcR,n=2L⋅Re{Tr{Rx¯−1A¯˙cR,mRsA¯HRx¯−1A¯˙cR,nRsA¯H}+Tr{Rx¯−1A¯˙cR,mRsA¯HRx¯−1A¯RsA¯˙cR,nH}}

(A7) FcI,mcI,n=2L⋅Re{Tr{Rx¯−1A¯˙cI,mRsA¯HRx¯−1A¯˙cI,nRsA¯H}+Tr{Rx¯−1A¯˙cI,mRsA¯HRx¯−1A¯RsA¯˙cI,nH}}

(A8) FcR,mcI,n=2L⋅Re{Tr{Rx¯−1A¯˙cR,mRsA¯HRx¯−1A¯˙cI,nRsA¯H}+Tr{Rx¯−1A¯˙cR,mRsA¯HRx¯−1A¯RsA¯˙cI,nH}}

(A9) $$F_{\rho \rho }=L\cdot ({\bar{A}}^{H}R_{\bar{x}}^{-1}\bar{A})⊙{\left({\bar{A}}^{H}R_{\bar{x}}^{-1}\bar{A}\right)}^{T}$$

(A10) Fθφ=2L⋅Re{(RsA¯HRx¯−1A¯˙θ)⊙(RsA¯HRx¯−1A¯˙φ)T+(RsA¯HRx¯−1A¯Rs)⊙(A¯˙φHRx¯−1A¯˙θ)T}

(A11) Fϕφ=2L⋅Re{(RsA¯HRx¯−1A¯˙ϕ)⊙(RsA¯HRx¯−1A¯˙φ)T+(RsA¯HRx¯−1A¯Rs)⊙(A¯˙φHRx¯−1A¯˙ϕ)T}

(A12) FθcR,n=2L⋅Re{diag{RsA¯HRx¯−1A¯˙cR,nRsA¯HRx¯−1A¯˙θ}+diag{RsA¯HRx¯−1A¯RsA¯˙cR,nHRx¯−1A¯˙θ}}

(A13) FϕcR,n=2L⋅Re{diag{RsA¯HRx¯−1A¯˙cR,nRsA¯HRx¯−1A¯˙θ}+diag{RsA¯HRx¯−1A¯RsA¯˙cR,nHRx¯−1A¯˙θ}}

(A14) FφcR,n=2L⋅Re{diag{RsA¯HRx¯−1A¯˙cR,nRsA¯HRx¯−1A¯˙φ}+diag{RsA¯HRx¯−1A¯RsA¯˙cR,nHRx¯−1A¯˙φ}}

(A15) FθcI,n=2L⋅Re{diag{RsA¯HRx¯−1A¯˙cI,nRsA¯HRx¯−1A¯˙θ}+diag{RsA¯HRx¯−1A¯RsA¯˙cI,nHRx¯−1A¯˙θ}}

(A16) FϕcI,n=2L⋅Re{diag{RsA¯HRx¯−1A¯˙cI,nRsA¯HRx¯−1A¯˙ϕ}+diag{RsA¯HRx¯−1A¯RsA¯˙cI,nHRx¯−1A¯˙ϕ}}

(A17) FφcI,n=2L⋅Re{diag{RsA¯HRx¯−1A¯˙cI,nRsA¯HRx¯−1A¯˙φ}+diag{RsA¯HRx¯−1A¯RsA¯˙cI,nHRx¯−1A¯˙φ}}

(A18) Fθρ=2L⋅Re{(RsA¯HRx¯−1A¯)⊙(A¯HRx¯−1A¯˙θ)T}

(A19) Fϕρ=2L⋅Re{(RsA¯HRx¯−1A¯)⊙(A¯HRx¯−1A¯˙ϕ)T}

(A20) Fφρ=2L⋅Re{(RsA¯HRx¯−1A¯)⊙(A¯HRx¯−1A¯˙φ)T}

(A21) FcR,m,ρn=2L⋅Re{a¯nHRx¯−1A¯˙cR,mRsA¯HRx¯−1a¯n}

(A22) FcI,m,ρn=2L⋅Re{a¯nHRx¯−1A¯˙cI,mRsA¯HRx¯−1a¯n}

where the partial derivative of A with respect to the parameters $\theta ,\phi ,\varphi ,c_{R}^{},c_{I}^{}$ in the above equations can be calculated as:

(A23) $${\dot{\bar{A}}}_{\theta }=\left[\frac{\partial {\bar{a}}_{1}}{\partial \theta_{1}},\cdots ,\frac{\partial {\bar{a}}_{K}}{\partial \theta_{K}}\right],\;{\dot{\bar{A}}}_{\phi }=\left[\frac{\partial {\bar{a}}_{1}}{\partial \phi_{1}},\cdots ,\frac{\partial {\bar{a}}_{K}}{\partial \phi_{K}}\right]$$

(A24) $${\dot{\bar{A}}}_{\varphi }=\left[\frac{\partial {\bar{a}}_{1}}{\partial \varphi_{1}},\cdots ,\frac{\partial {\bar{a}}_{K}}{\partial \varphi_{K}}\right]$$

(A25) $${\dot{\bar{A}}}_{c_{R,}{}_{m}}=\left[\begin{array}{c} \frac{\partial C}{\partial c_{R,}{}_{m}}A\Phi \\ \frac{\partial C^{*}}{\partial c_{R,}{}_{m}}A^{*}\Phi^{*} \end{array}\right],\;{\dot{\bar{A}}}_{c_{I,}{}_{m}}=\left[\begin{array}{c} \frac{\partial C}{\partial c_{I,}{}_{m}}A\Phi \\ \frac{\partial C^{*}}{\partial c_{I,}{}_{m}}A^{*}\Phi^{*} \end{array}\right]$$

Since the noise power is not coupled with other parameters, the cross terms with respect to $\sigma_{n}^{2}$ are all zeros. Consequently, the FIM can be formulated as the following form:

(A26) $$F=\left[\begin{array}{lllllll} F_{\theta \theta } & F_{\theta \phi } & F_{\theta \varphi } & F_{\theta c_{r}} & F_{\theta c_{I}} & F_{\theta \rho } & 0\\ F_{\phi \theta } & F_{\phi \phi } & F_{\phi \varphi } & F_{\phi c_{r}} & F_{\phi c_{I}} & F_{\phi \rho } & 0\\ F_{\varphi \theta } & F_{\varphi \phi } & F_{\varphi \varphi } & F_{\varphi c_{r}} & F_{\varphi c_{I}} & F_{\varphi \rho } & 0\\ F_{c_{r}\theta } & F_{c_{r}\phi } & F_{c_{r}\varphi } & F_{c_{r}c_{r}} & F_{c_{r}c_{I}} & F_{c_{r}\rho } & 0\\ F_{c_{I}\theta } & F_{c_{I}\phi } & F_{c_{I}\varphi } & F_{c_{I}c_{r}} & F_{c_{I}c_{I}} & F_{c_{I}\rho } & 0\\ F_{\rho \theta } & F_{\rho \phi } & F_{\rho \varphi } & F_{\rho c_{r}} & F_{\rho c_{I}} & F_{\rho \rho } & 0\\ 0 & 0 & 0 & 0 & 0 & 0 & F_{\sigma_{n}^{2}\sigma_{n}^{2}} \end{array}\right]$$

Subsequently, the CRB for DOA parameters is defined as:

(A27) $$CRB_{DOA}=\sqrt{\frac{1}{2K}\sum_{k=1}^{2K}{\left[F^{-1}\right]}_{kk}}$$
